# The impact of preoperative choroidal detachment on short-term surgical outcomes in highly myopic macular hole retinal detachment

**DOI:** 10.3389/fmed.2025.1662026

**Published:** 2025-10-06

**Authors:** Yi Cai, Jia Liu, Xiaoxin Li, Mingwei Zhao, Jianhong Liang, Hong Yin, Wenzhen Yu, Huijun Qi, Tong Qian, Xuan Shi, Jinfeng Qu, Yong Cheng, Jing Hou, Enzhong Jin, Heng Miao

**Affiliations:** ^1^Department of Ophthalmology, Peking University People's Hospital, Beijing, China; ^2^Beijing Key Laboratory of Ocular Disease and Optometry Science, Peking University People’s Hospital, Beijing, China

**Keywords:** macular hole retinal detachment, choroidal detachment, highly myopic, pars plana vitrectomy, prognosis

## Abstract

**Purpose:**

To evaluate the impact of choroidal detachment on short-term surgical outcomes in highly myopic macular hole retinal detachment (MHRD) and to discern the risk factors associated with macular hole (MH) closure.

**Methods:**

A retrospective analysis was carried out on 104 highly myopic MHRD eyes that underwent vitrectomy and intraocular tamponade. Patients were stratified according to the presence or absence of preoperative choroidal detachment. Demographic data, preoperative ocular parameters, and postoperative outcomes were compared. Logistic regression analysis was employed to identify risk factors for MH closure.

**Results:**

No significant differences were observed in age or disease duration between groups. Preoperative visual acuity was inferior in the choroidal detachment group (2.19 ± 0.70 LogMAR *vs.* 1.78 ± 0.60 LogMAR, *p* = 0.013), and preoperative intraocular pressure (IOP) was significantly lower (9.05 ± 3.73 mmHg *vs.* 13.18 ± 4.15 mmHg, *p* < 0.001). Postoperatively, the choroidal detachment group demonstrated worse vision outcome (1.52 ± 0.49 LogMAR *vs*. 1.22 ± 0.38 LogMAR, *p* = 0.004) and a substantially higher rate of rescue therapy (28.6% *vs.* 3.6%, *p* = 0.002). The rate of MH closure was comparable between groups (41.0% *vs.* 42.9%, *p* = 1.000). Logistic regression identified advanced age (*β* = 0.099, *p* = 0.001) and lower preoperative IOP (β = −0.132, *p* = 0.031) as favorable factors for MH closure.

**Conclusion:**

Choroidal detachment exerts an adverse impact on postoperative visual acuity and is associated with a significantly higher rate of rescue surgical intervention. Younger age and higher preoperative intraocular pressure (IOP) may impede MH closure.

## 1 Introduction

Macular hole retinal detachment (MHRD) is one of the most vision-threatening complications associated with high myopia and has emerged as a significant factor impacting visual function and quality of life in patients with severe myopia, particularly given the rising prevalence of myopia globally (1, 2). Although the pathogenesis of MHRD remains incompletely understood, it is believed to involve both pre-retinal and sub-retinal factors, making surgical management particularly challenging in highly myopic MHRD eyes (3, 4).

Currently, the optimal surgical approach for MHRD remains a subject of debate. Various techniques, including pars plana vitrectomy (PPV) combined with gas or silicone oil tamponade, macular buckling, and extramacular drainage, have been explored to improve anatomical and functional outcomes. Nevertheless, PPV accompanied by internal limiting membrane (ILM) peeling has emerged as the most widely adopted surgical procedure (5–8). MHRD remains one of the most challenging types of retinal detachment due to the loss of choroidal capillaries, which reduces adhesion between the retina and retinal pigment epithelium (RPE), increasing the risk of recurrent detachment (3, 8).

Rhegmatogenous Retinal Detachment (RRD) associated with choroidal detachment, first described by Gottlieb et al. in 1974 (9), is a rare but severe variant more commonly observed in Asian populations. This condition is often accompanied by intraocular inflammatory signs, including corneal edema, anterior chamber reaction, posterior synechiae, and vitreous opacity (9, 10). It is characterized by rapid progression, poor prognosis, and significant treatment challenges, with an incidence ranging from 2 to 4.5% among all RRD cases (11). The pathological process involves retinal tears, retinal detachment, choroidal detachment, and low intraocular pressure (IOP), with proliferative vitreoretinopathy (PVR) playing a pivotal role in surgical failure (12).

Despite these challenges, limited research has focused on the surgical outcomes and prognosis of MHRD in highly myopic eyes with choroidal detachment. This retrospective cohort study aimed to compare the preoperative and postoperative characteristics and features, as well as short-term surgical outcomes of MHRD in highly myopic patients with and without choroidal detachment, and to identify risk factors influencing macular hole (MH) closure.

## 2 Methods

### 2.1 Study design

This retrospective, institution-based cohort study involving high myopia macular hole retinal detachment (MHRD) patients was approved by the Ethical Review Committee of Peking University People’s Hospital (Beijing, China), which was conducted in accordance with the Declaration of Helsinki. Written informed consent was obtained from each patient prior to surgery. All included patients underwent 25-gauge pars plana vitrectomy (PPV) combined with silicone oil or gas tamponade between January 2019 and June 2024 at the Department of Ophthalmology, Peking University People’s Hospital. Patients were followed for a minimum duration of 6 months post-primary vitrectomy. The highly myopic MHRD eyes were categorized into two groups based on the presence or absence of choroidal detachment. Demographic characteristics, preoperative ocular conditions, and surgical outcomes were subsequently compared between the two groups.

### 2.2 Inclusion criteria and exclusion criteria

Inclusion criteria were as follows: highly myopic eyes exhibiting an axial length ≥ 26.5 mm or cycloplegic spherical equivalent ≤−6.00 D (1, 13); diagnosis of MHRD necessitating PPV combined with silicone oil or gas tamponade; minimum follow-up duration of 3 months.

Exclusion criteria encompassed: previous intraocular surgeries (excluding cataract surgery performed more than 12 months prior to presenting with MHRD); choroidal or retinal detachments secondary to ocular tumors, uveitis, or other etiologies; non-highly myopic MHRD; traumatic injuries or other ocular diseases predisposing to secondary MHRD.

### 2.3 Surgical procedure

For patients presenting with choroidal detachment, a preoperative anti-inflammatory protocol was administered. This consisted of a peribulbar injection of 20 mg triamcinolone acetonide, performed within 3 days prior to the scheduled vitrectomy. All PPV procedures were performed by experienced vitreoretinal surgeons utilizing a standard 25-gauge three-port system (Constellation, Alcon, USA), under retrobulbar anesthesia or general anesthesia. External drainage of subchoroidal fluid was performed at the surgeon’s discretion for moderate-to-severe choroidal detachment, typically in cases with ‘kissing’ choroidals or extensive bullous choroidal detachment that impeded safe intraocular instrumentation. Suprachoroidal puncture was performed near the uppermost part of the detachment through a pars plana sclerotomy during vitrectomy. Concurrent cataract surgery with intraocular lens implantation was conducted when indicated. Core and peripheral vitreous were thoroughly removed, and triamcinolone acetonide was used to enhance visualization of residual vitreous or proliferative membranes, which were gently excised using intraocular forceps. Internal limiting membrane (ILM) peeling was performed within the vascular arcade after staining with indocyanine green (ICG) for 60 s. Extended-length instrumentation was used as required for eyes with high axial myopia to ensure safe and effective access to the macula. After ILM peeling, a fluid-air exchange was conducted, and peripheral SRF was first drained through any preexisting peripheral retinal breaks. Subsequently, the remaining fluid at the posterior pole was carefully drained internally through the macular hole using a soft-tip flute needle, then followed by tamponade with silicone oil or long-acting gas. To ensure a homogeneous surgical cohort and avoid confounding variables from different macular hole repair techniques, this study exclusively included cases where a standard ILM peeling technique was used. Cases utilizing inverted ILM flap techniques or amniotic membrane grafts were excluded from this analysis. Patients were instructed to maintain a face-down position for 1 month postoperatively (14). Silicone oil removal was performed at least 3 months after tamponade.

### 2.4 Structural and functional examination

All patients underwent comprehensive evaluations 1 day before surgery and at day one, week one, and month three after operation. Assessments included best-corrected visual acuity (BCVA), slit-lamp examination, non-contact intraocular pressure (IOP), indirect ophthalmoscopy, ultrawide fundus photography and optical coherence tomography (OCT). The extent of choroidal detachment was categorized into 1–4 quadrants based on B-scan ultrasound and intraoperative findings. BCVA was measured using Snellen charts and converted to the logarithm of the minimal angle of resolution (LogMAR) for analysis. Finger counting and hand motion were assigned LogMAR values of 1.8 and 3, respectively. Preoperative proliferative vitreoretinopathy (PVR) was graded based on a review of fundus photographs and clinical records. Due to the limitations of retrospective data, PVR was categorized into three groups for analysis: (1) No PVR: no evidence of any proliferative membranes; (2) Mild-to-moderate PVR: presence of epiretinal membranes or localized retinal traction; and (3) Severe PVR: extensive proliferative membranes involving the entire retina. Postoperative OCT (OCT, Cirrus HD-OCT 5000 AngioPlex, Carl Zeiss Meditec, Germany) was performed at month three to assess retinal reattachment, MH closure, and central foveal structure integrity. Successful retinal reattachment was defined as complete absorption of subretinal fluid and full neuroretina adhesion to the RPE without foveal margin elevation. Macular hole (MH) closure was defined as the absence of neuroretinal tissue loss at the macular fovea on OCT. Axial length (AL) was measured 3 months postoperatively using the IOL Master 700 (Carl Zeiss Meditec, Germany). Quantitative data were measured three times to obtain an average value.

### 2.5 Statistical analysis

Statistical analysis was conducted using RStudio (Version 1.4.1717). Continuous variables were expressed as mean ± standard deviation (SD) and compared using independent *t*-tests, Welch’s *t*-tests, or Mann–Whitney *U* tests, depending on normality and variance homogeneity. Categorical variables were summarized as frequencies and percentages and compared using chi-square or Fisher’s exact tests. Multiple linear regression and logistic regression were employed to identify factors associated with postoperative MH closure. A *p* value < 0.05 was considered statistically significant, and post-hoc pairwise comparisons with Bonferroni correction were performed to identify overall significance.

## 3 Results

### 3.1 Demographic and ocular parameters of the participants

A total of 104 highly myopic MHRD eyes from 100 participants were included in this study. The mean age of the cohort was 60.88 ± 9.32 years, with 79 (79.0%) participants being female. Choroidal detachment was observed in 21 (20.2%) eyes, with the extent of choroidal detachment varying: two eyes (1.9%) exhibited involvement in one quadrant, three eyes (2.9%) in two quadrants, three eyes (2.9%) in three quadrants, and the remaining 13 eyes (12.5%) demonstrated involvement across all four quadrants. Severe kissing choroidal detachment was observed in three eyes (2.9%). The average axial length (AL) measured postoperatively at month three was 29.51 ± 2.30 mm.

**Figure 1:**
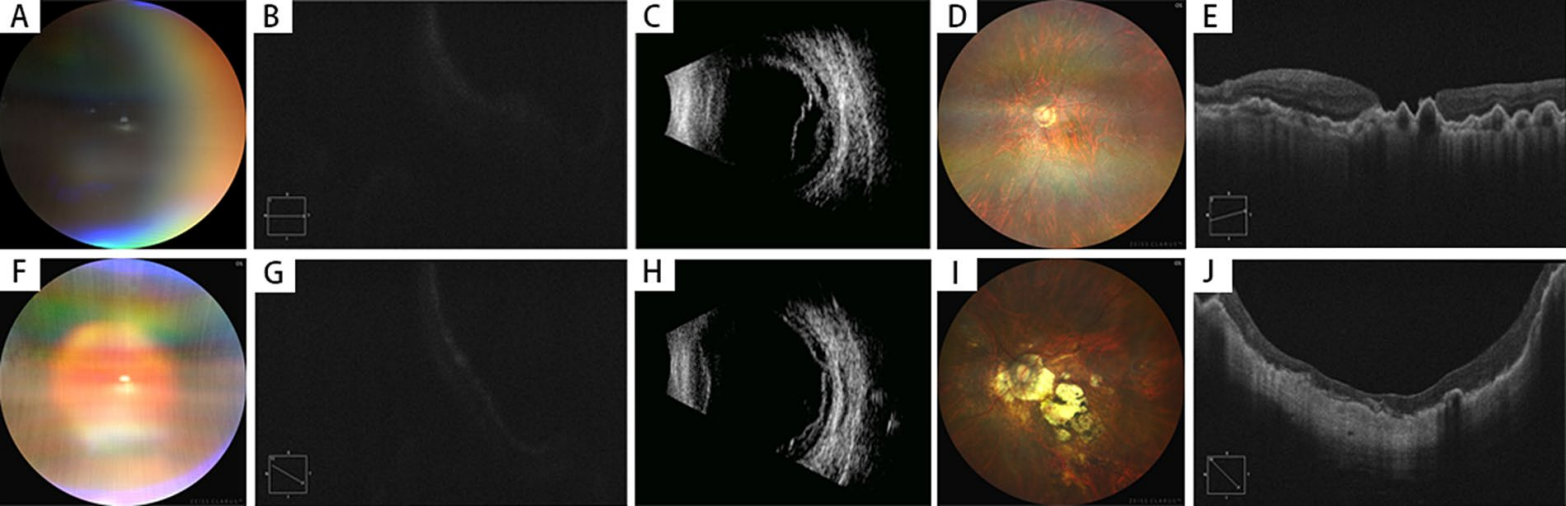
Preoperative and postoperative examinations of highly myopic eyes with macular hole retinal detachment (MHRD) with choroidal detachment (2 Cases). **(A–E)** Case 1 (MH Unclosed)–A 67-year-old female presented with 2 years of vision loss in her left eye, diagnosed as MHRD with choroidal detachment **(A)**. Preoperative best-corrected visual acuity (BCVA) was 0.04 (LogMAR 1.4), and OCT could not clearly delineate the macular hole **(B)**. B-scan ultrasonography confirmed the presence of both retinal and choroidal detachments **(C)**. Postoperatively, the retina was reattached under silicone oil tamponade **(D)**, but the macular hole remained open with a diameter of 1,005 μm **(E)**. The final postoperative BCVA was 0.04 (LogMAR 1.4), and the axial length measured 29.56 mm. J: Case 2 (MH Closed)–A 64-year-old female presented with 20 days of vision loss in her left eye, also diagnosed as MHRD with choroidal detachment **(F)**. Preoperative BCVA was counting fingers (LogMAR 1.8), and OCT imaging was obscured **(G)**. B-scan ultrasonography confirmed retinal and choroidal detachments **(H)**. The patient underwent an initial vitrectomy with silicone oil tamponade, but developed a recurrent retinal detachment postoperatively. A secondary surgical intervention was performed, after which the retina was successfully reattached **(I)**, and complete macular hole closure was achieved, with a CFT of 86 μm **(J)**. The final postoperative BCVA improved to 0.05 (LogMAR 1.3), and the axial length measured 27.6 mm.

**Figure 2:**
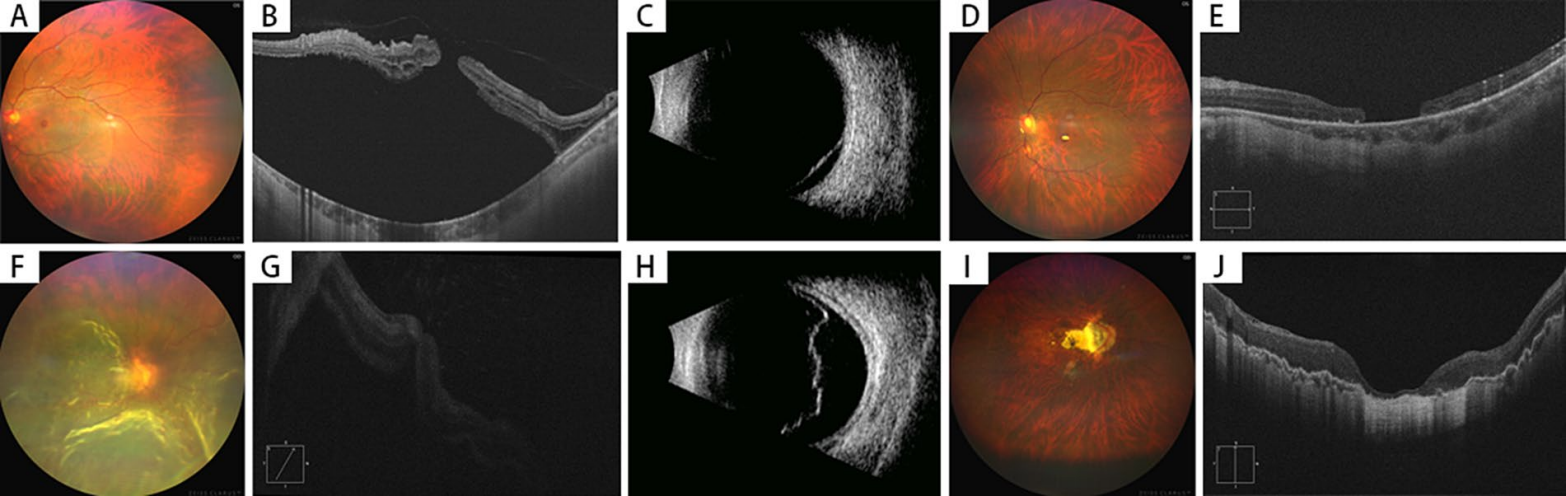
Preoperative and postoperative examinations of highly myopic eyes with macular hole retinal detachment (MHRD) without choroidal detachment (2 Cases). **(A–E)** Case 1 (MH Unclosed)–A 64-year-old female presented with 4 years of vision loss in her left eye, diagnosed as MHRD without choroidal detachment **(A)**. Preoperative BCVA was 0.02 (LogMAR 1.7), and the macular hole could be noticed on OCT with extensive subretinal fluid **(B)**. B-scan ultrasonography confirmed retinal detachment **(C)**. Postoperatively, while the retina was reattached under silicone oil tamponade **(D)**, the macular hole failed to close completely, with a diameter of 967 μm **(E)**. The postoperative BCVA was 0.03 (LogMAR 1.5), and the axial length measured 26.5 mm. **(F–J)** Case 2–A 60-year-old male presented with 20 days of vision loss in the right eye was diagnosed with MHRD without choroidal detachment **(F)**. Preoperative BCVA: counting fingers (LogMAR 1.8); OCT showed approximation of the macular hole edges, although visualization was limited due to poor image quality **(G)**. Ultrasound confirmed retinal detachment **(H)**. Intraoperatively, peripheral retinal tears and MH without choroidal detachment were observed. Postoperatively, retinal reattachment was achieved, and MH was closed, with a CRT of 57 μm **(J)**. The AL measured 29.1 mm with silicone oil tamponade, while postoperative BCVA improved to 0.2 (LogMAR 0.7).

Clinical features were compared between MHRD patients with (choroidal detachment group) and without (non-choroidal detachment group) choroidal detachment (Table 1 and Figures 1, 2). Mean age (61.90 ± 10.57 years *vs.* 60.61 ± 9.01 years, *p* = 0.406) and average disease duration prior to surgery (167 ± 446 days *vs.* 119 ± 256 days, *p* = 0.567) were comparable between the two groups. No significant differences were observed in laterality or gender distribution, either. Preoperative best corrected vision acuity (BCVA) was significantly inferior in the choroidal detachment group compared to the non-choroidal detachment group (1.52 ± 0.49 LogMAR vs. 1.22 ± 0.38 LogMAR, *p* = 0.004). Additionally, the preoperative IOP in the choroidal detachment group was markedly lower at 9.05 ± 3.73 mmHg compared to 13.18 ± 4.15 mmHg in the non-choroidal detachment group (*p* < 0.001). Analysis of preoperative PVR revealed a significant association between PVR severity and the presence of choroidal detachment (Fisher’s exact test, *p* = 0.007). Patients in the choroidal detachment group presented with a more severe distribution of PVR grades compared to the non-choroidal detachment group. Specifically, 14.3% of patients in the choroidal detachment group had severe PVR, whereas 0% of patients in the non-choroidal detachment group had severe PVR. Post-hoc pairwise comparisons with Bonferroni correction were performed to identify the source of this overall significance. The analysis confirmed a significant difference in the distribution between the no PVR and severe PVR categories (adjusted *p* = 0.021). In contrast, no significant differences were found between the no PVR and mild-to-moderate PVR categories (adjusted *p* = 1.00) or between the mild-to-moderate PVR and severe PVR categories (adjusted *p* = 0.43). To mitigate the confounding effects of recent intraocular surgery, we specifically reviewed the surgical history of all pseudophakic patients. All patients who had previously undergone cataract surgery had their procedure performed more than 1 year with a stable postoperative course, prior to the presentation of the symptom of MHRD.

**Table 1:** Baseline clinical characteristics and postoperative outcomes in highly myopic macular hole retinal detachment patients with and without choroidal detachment.

Characteristics	MHRD without choroidal detachment	MHRD with choroidal detachment	Total	*P-*value
Gender				0.371
Male (*n*, %)	15 (19.0%)	6 (28.6%)	21 (21.0%)	
Female (*n*, %)	64 (81.0%)	15 (71.4%)	79 (79.0%)	
Age (years, mean ± SD)	60.61 ± 9.01	61.90 ± 10.57	60.88 ± 9.32	0.406
Included eyes (*n*)	*n* = 83	*n* = 21	*n* = 104	
Axial length (mm, mean ± SD)	29.49 ± 2.42	29.61 ± 1.83	29.51 ± 2.30	0.490
Duration of disease (days, mean ± SD)	119.07 ± 256.23	167.24 ± 445.76	128.99 ± 302.87	0.567
Preoperative BCVA (logMAR, mean ± SD)	1.78 ± 0.60	2.19 ± 0.70	1.86 ± 0.64	**0.013**
Preoperative IOP (mmHg, mean ± SD)	13.18 ± 4.15	9.05 ± 3.73	12.34 ± 4.38	**<0.001**
Postoperative BCVA (logMAR, mean ± SD)	1.22 ± 0.38	1.52 ± 0.49	1.28 ± 0.42	**0.030**
Improvement of BCVA (LogMAR, mean ± SD)	−0.56 ± 0.64	−0.67 ± 0.73	−0.58 ± 0.66	0.490
Preoperative PVR				**0.007**
No significant PVR (*n*, %)	80 (96.4%)	17 (81.0%)	97 (93.3%)	
Mild-to-moderate PVR (*n*, %)	3 (3.6%)	1 (4.8%)	4 (3.8%)	
Severe PVR (*n*, %)	0 (0%)	3 (14.3%)	3 (2.9%)	
Number of operations				**0.002**
1 (*n*, %)	80 (96.4%)	15 (71.4%)	60 (88.2%)	
2 (*n*, %)	3 (3.6%)	5 (23.8%)	7 (10.3%)	
3 (*n*, %)	0 (0%)	1 (4.8%)	1 (1.5%)	
Introcular tamponade				
Gas (13% C_3_F_8_) (%)	3 (3.6%)	0 (0%)	3 (2.9%)	
Silicon oil (%)	80 (96.4%)	21 (100%)	101 (97.1%)	
Postoperative MH closure rate (*n*, %)	34 (41.0%)	9 (42.9%)	43 (41.3%)	1.000

MHRD: Macular hole retinal detachment; BCVA: Best corrected visual acuity; IOP: Intraocular pressure; LogMAR: Logarithm of the minimum angle of resolution; MH: Macular hole; PVR: proliferative vitreoretinopathy; SD: Standard Deviation; N: number of cases. Bold: *p* < 0.05.

### 3.2 Surgical outcomes of highly myopic MHRD eyes

Considering the complexity of highly myopic MHRD, silicone oil was the preferred tamponade agent in the vast majority of cases (101/104, 97.1%). Long-acting (13% C_3_F_8_) gas tamponade was reserved for three cases with less extensive retinal detachment and favorable peripheral retinal conditions in the non-choroidal detachment group. At 3 months postoperatively, the mean BCVA in the choroidal detachment group improved to 1.52 ± 0.49 LogMAR (*P* < 0.001) but remained inferior to that of the non-choroidal detachment group (1.22 ± 0.38 LogMAR, *p* = 0.002). However, there was no significant difference in the improvement of BCVA between the two groups (−0.67 ± 0.73 LogMAR *vs.* –0.56 ± 0.64 LogMAR, *p* = 0.490). Furthermore, no significant discrepancy was noted concerning AL measurements taken postoperatively at month three following retinal reattachment between the groups (29.61 ± 1.83 mm *vs*. 29.49 ± 2.42 mm, *p* = 0.490).

### 3.3 Choroidal detachment elevates the risk of requiring rescue surgery

Three months after primary operation, eyes with choroidal detachment were more likely to require rescue surgery than eyes without (28.6% *vs.* 3.6%, *p* = 0.002) due to failed retinal reattachment or recurrent retinal detachment. However, there was no significant difference in terms of the MH closure rate between the two groups (42.9% *vs*. 41.0%, *p* = 1.000).

### 3.4 Younger age and higher preoperative IOP impede MH closure

Three months after surgery, MH closure was achieved in 43 eyes (41.3%), while 61 eyes (58.7%) exhibited unclosed. The average diameter of unclosed MHs was 809.87 ± 404.93 μm. No significant differences were observed in disease duration, postoperative AL, BCVA 3 months after surgery, or BCVA improvement between the closed and unclosed MH groups (*p* = 0.546, *p* = 0.554, *p* = 0.840, and *p* = 0.722, respectively) (Table 2). However, patients in the MH closure group were markedly older (64.17 ± 8.65 years) compared to the unclosed group (58.59 ± 9.14 years, *p* = 0.001). The extent of choroidal detachment did not exhibit a statistically significant association with MH closure (*p* = 0.256). Logistic regression analysis revealed that younger age (*β* = 0.100, *P* < 0.001) and higher preoperative IOP (β = −0.132, *p* = 0.031) were significantly associated with unclosed MHs, while disease duration, preoperative BCVA, presence of choroidal detachment, extent of PVR, and AL 3 months after surgery were not.

**Table 2:** Risk factors for postoperative unclosed macular hole in highly myopic macular hole retinal detachment patients.

Characteristics	MHRD with unclosed MH post-operative	MHRD with closed MH post-operative	Total	*p-*value	Logistic analysis	
					*β*	*p*-value
Gender				0.956		
Male (*n*, %)	13 (22.0%)	8 (19.5%)	21 (21.0%)			
Female (*n*, %)	46 (78.0%)	33 (80.5%)	79 (79.0%)			
Age (years, mean ± SD)	58.59 ± 9.14	64.17 ± 8.65	60.88 ± 9.32	0.001	0.098	**0.001**
Included eyes (*n*)	*n* = 61	*n* = 43	*n* = 104			
Axial length (mm, mean ± SD)	29.64 ± 2.36	29.33 ± 2.24	29.51 ± 2.30	0.554	−0.020	0.850
Duration of disease (days, mean ± SD)	146.34 ± 313.99	103.17 ± 287.39	128.99 ± 302.87	0.546	−0.001	0.450
Choroidal detachment				1.000	−0.620	0.308
Positive (*n*, %)	12 (19.7%)	9 (20.9%)	21 (20.2%)			
Negative (*n*, %)	49 (80.3%)	34 (79.1%)	83 (79.8%)			
Preoperative PVR				0.844		
No significant PVR (*n*, %)	56 (91.8%)	41 (95.3%)	97 (93.3%)			
Mild-to-moderate PVR (*n*, %)	3 (4.9%)	1 (2.3%)	4 (3.8%)			
Severe PVR (*n*, %)	2 (3.3%)	1 (2.3%)	3 (2.9%)			
Preoperative BCVA (logMAR, mean ± SD)	1.74 ± 0.41	1.77 ± 0.51	1.76 ± 0.45	0.814	0.494	0.193
Preoperative IOP (mmHg, mean ± SD)	12.95 ± 4.48	11.48 ± 4.12	12.34 ± 4.38	0.103	−0.132	**0.031**
Postoperative BCVA (logMAR, mean ± SD)	1.23 ± 0.34	1.34 ± 0.48	1.28 ± 0.41	0.840		
Improvement of BCVA (LogMAR, mean ± SD)	−0.51 ± 0.48	−0.43 ± 0.51	−0.48 ± 0.49	0.722		
Introcular tamponade				1.000		
Gas (13% C_3_F_8_) (%)	2 (3.3%)	1 (2.3%)	3 (2.9%)			
Silicon oil (%)	59 (96.7%)	42 (97.7%)	101 (97.1%)			

MHRD: Macular hole retinal detachment; MH: Macular hole; BCVA: Best corrected visual acuity; IOP: Intraocular pressure; LogMAR: Logarithm of the minimum angle of resolution; SD: Standard Deviation; N: number of cases. Bold: *p* < 0.05.

## 4 Discussion

The pathogenesis and prognostic factors of highly myopic MHRD remain incompletely understood, with limited clinical research specifically addressing MHRD associated with choroidal detachment. The present study aimed to compare demographic and preoperative characteristics, postoperative clinical manifestations, and surgical outcomes in patients with highly myopic MHRD, stratified by the presence or absence of choroidal detachment. Our findings provide insights into the impact of choroidal detachment on postoperative visual outcome, MH closure rates, and the necessity for rescue surgery therapy in cases of MHRD.

Li et al. reported that patients with RRD accompanied by choroidal detachment tended to be older, but detailed statistical comparisons were not provided (15). In this study, our findings revealed no significant differences in demographic characteristics between patients with or without choroidal detachment. Similarly, other studies found no significant associations between MHRD with choroidal detachment and systemic factors such as gender, age, or comorbidities like hypertension and diabetes (16). The comparable demographic profiles in our study enhance the validity of inter-group comparisons.

The development of MHRD with choroidal detachment likely follows a complex pathological cascade. It has been proposed that in some highly myopic eyes, a posterior vitreous detachment (PVD) allows liquefied vitreous to pass through the macular hole (11), leading to progressive subretinal fluid accumulation. This process can disrupt the normal fluid dynamics across the retinal pigment epithelium (RPE) (16, 17), resulting in hypotony—which was verified in the finding in our choroidal detachment cohort (9.05 ± 3.73 mmHg) and other research (11, 17). This state of chronic hypotony and significant intraocular inflammation, often associated with CD, creates a potent pro-proliferative environment. These conditions are known to stimulate the epithelial-mesenchymal transition of RPE cells, which is the core cellular event in the pathogenesis of PVR. Our study’s findings provide clinical evidence supporting this pathophysiological link. We identified a significant association between the presence of choroidal detachment and a more severe grade of preoperative PVR (Fisher’s exact test, *p* = 0.007). The post-hoc analysis confirmed that this was driven by a significantly higher proportion of severe PVR cases in the choroidal group. This heightened PVR burden offers a direct explanation for several of our other key findings. Firstly, it likely accounts for the significantly worse preoperative BCVA observed in the choroidal detachment group. More critically, it provides a mechanistic explanation for the disparity in surgical outcomes, as the choroidal detachment group required a significantly higher rate of further surgical interventions (28.6% vs. 3.6%, *p* = 0.002).

In highly myopic eyes with MHRD, persistent hypotony can cause dilation of choroidal vessels, leading to continuous fluid leakage into the choroidal cavity and exacerbating choroidal detachment. This mechanism suggests that disease duration might be a risk factor for choroidal detachment in RRD. However, our study found no statistically significant differences in disease duration between the groups with and without choroidal detachment, which was consistent with previous studies (12, 16). This indicates that disease duration may not significantly impact visual outcomes in MHRD complicated by choroidal detachment. The lack of association could be attributed to discrepancies between actual and recorded disease durations. Further prospective studies are needed to address this issue.

All patients in the choroidal detachment group received silicone oil tamponade, while three patients in the non-choroidal detachment group underwent gas (13% C_3_F_8_) tamponade. The rescue surgery rate was significantly higher in the choroidal detachment group (28.6% vs. 3.6%, *p* = 0.002), despite the use of silicone oil, which is generally associated with greater stability. This finding aligns with previous studies indicating that highly myopic MHRD with choroidal detachment often involves larger retinal detachments, multiple tears, and advanced PVR, all of which increase the risk of surgical failure (10). Additionally, choroidal and RPE atrophy, combined with tangential traction from posterior scleral staphyloma, further complicates surgery and increases the risk of recurrence of retinal detachment (18, 19). To address these complexities, the controlled drainage of subchoroidal fluid is a critical maneuver that serves to mitigate intraoperative risks and facilitate subsequent surgical steps (20). The rationale for performing external subchoroidal fluid drainage in these complex cases is twofold, addressing both postoperative stability and intraoperative safety. Postoperatively, adequate drainage is essential to accommodate a sufficient volume of silicone oil tamponade. This preemptively counteracts the risk of subsequent hypotony and compromised retinal support that can arise from a relative underfill as the choroid reattaches. Intraoperatively, the procedure is critical for securing safe cannula placement. A detached choroid significantly increases the risk of inadvertent infusion into the suprachoroidal space—a severe complication that can precipitate massive choroidal expansion, hemorrhage, and iatrogenic retinal breaks. Therefore, controlled external drainage is a key step in simplifying the surgical field and minimizing procedural risks (20, 21).

At 3 months postoperatively, BCVA in the choroidal detachment group remained significantly worse than in the non-choroidal detachment group, suggesting that choroidal detachment negatively impacts postoperative visual outcomes. This may be attributed to inflammation hindering neuroretina recovery (19). However, there was no significant discrepancy in MH closure rates between the two groups, indicating that choroidal detachment does not directly influence MH closure. Following surgery for idiopathic MHs, inward migration of retinal inner layers and photoreceptor cells facilitates reformation of the macular fovea, a process thought to correlate with improved vision. Consequently, previous studies on treating highly myopic MHRD have speculated that successful postoperative MH closure could potentially enhance visual outcomes. However, the relationship between anatomical closure and functional improvement in highly myopic MHRD remains less clear, highlighting the need for further investigation into the underlying mechanisms and prognostic factors.

Pars plana vitrectomy combined with ILM peeling has been a standard surgical approach for MHRD over the past decade, which effectively alleviates tangential retinal traction. However, postoperative MH closure rates remain suboptimal, ranging from 10 to 70% (7). In this study, complete MH closure was achieved in 43 out of 104 eyes (41.3%) at the 3-month postoperative follow-up, consistent with rates reported in previous studies (22, 23). A similar rate of 43% for U-shaped and V-shaped MH closures following ILM peeling alone has been reported previously (22), while Chen et al. documented a closure rate of 42.9% after surgery (23). These findings suggest that PPV combined with ILM peeling alone does not achieve high anatomical closure rate in highly myopic MHRD. To address this limitation, adjunctive surgical techniques such as ILM inversion, covering techniques, autologous neuroretina transplantation, and amniotic membrane transplantation have been proposed to improve postoperative MH closure rates (7, 24). Despite these advancements, significant improvements in visual outcomes have not been consistently observed. Studies by Ikuno et al. (25) and Kim et al. (26) found no correlation between long-term visual outcomes and MH closure in highly myopic MHRD, a finding supported by our study, which showed no significant differences in postoperative BCVA or BCVA improvement between eyes with closed and unclosed MHs. In highly myopic eyes exhibiting postoperative MH closure, glial tissue often replaces neuroretinal tissue and covers exposed RPE (27). However, the normal outer retinal layer structure is not restored, and new choroidal or retinal capillary networks do not form, limiting functional visual improvement. Postoperative optical coherent tomography (OCT) frequently reveals excessive glial proliferation, characterized by high reflectivity and abnormal migration patterns, leading to retinal disorganization and irreversible photoreceptor damage (27). These findings highlight the inherent challenges in achieving both anatomical and functional success in highly myopic MHRD, emphasizing the need for further research into novel therapeutic strategies. On the other hand, our decision to include only ILM peeling cases was to isolate the impact of choroidal detachment without the confounding effect of varied surgical techniques. The presence of choroidal detachment, indicating a more severe disease state with pronounced chorioretinal atrophy, might pose additional challenges for ILM flap manipulation, such as flap stability and placement, though this requires further investigation.

The clinical factors influencing anatomical success in myopic MHRD surgery remain a subject of debate. A particularly noteworthy finding of our study is that younger age was a predictor of failed MH closure. This seemingly counterintuitive result is consistent with observations from Hong et al. and warrants a deeper exploration of the underlying pathophysiology (8). The leading hypothesis relates to the vitreoretinal interface in younger individuals. Although PVD is common in myopia, younger patients tend to have a more tenaciously adherent posterior vitreous cortex (28). Even after a thorough vitrectomy, microscopic vitreous remnants can persist on the retinal surface, exerting subtle but persistent tangential traction that impedes hole closure (28, 29). Furthermore, the biomechanical properties of the ILM in younger eyes, such as greater elasticity (30), and a stronger proliferative tissue response may also contribute to this phenomenon by promoting glial bridging rather than true retinal apposition (11, 31). In addition to age, our analysis identified higher preoperative IOP as another independent risk factor for non-closure, a finding that has been rarely reported and requires further investigation. Consistent with previous studies, however, we found no significant association between MH closure and other factors such as axial length, disease duration, or the presence of choroidal detachment (32, 33).

This study has several limitations. First, its retrospective design may introduce selection and recall biases. Future research should adopt a prospective design to address these limitations. Second, the sample size was relatively small and patients with choroidal detachment accounted for only about 20%, which may result in sample size imbalance and reduce the statistical power during comparison. Besides, the follow-up period was short, which may limit the generalizability of findings. Third, all patients included underwent traditional vitrectomy combined with ILM peeling and intraocular tamponade, precluding comparisons with alternative surgical techniques such as ILM coverage, inversion, or autologous blood tamponade. Undoubtedly, larger-scale studies with better-defined groups and extended follow-up periods are needed to further elucidate the impact of choroidal detachment on long-term surgical outcomes in highly myopic MHRD.

In conclusion, choroidal detachment in cases of highly myopic MHRD has a deleterious impact on both preoperative vision and postoperative visual acuity restoration, while significantly elevating the risk of recurrent retinal detachment and the requirement for rescue surgical intervention. Although choroidal detachment does not directly affect the rates of MH closure, younger age at disease onset and higher preoperative IOP are risk factors influencing postoperative MH closure.

## Data Availability

The raw data supporting the conclusions of this article will be made available by the authors, without undue reservation.
